# Paraduodenal hernia: An exceptional cause of acute bowel obstruction

**DOI:** 10.1002/ccr3.6886

**Published:** 2023-01-26

**Authors:** Med Mehdi Trabelsi, Annouar Oueslati, Mehdi Kammoun, Hichem Jerraya, Ibtissem Bouasker, Ramzi Nouira

**Affiliations:** ^1^ Department B of surgery Charles Nicolle Hospital Tunis Tunisia

**Keywords:** general surgery

## Abstract

Internal hernias represent only 0.2%–0.9% of all causes of bowel obstruction. A 59‐year‐old patient presented urgently with small bowel obstruction. Laparotomy revealed a left paraduodenal hernia with most of the small bowel herniating through a space between the inferior mesenteric vein and duodenojejunal junction.

## 1 DESCRIPTION

**FIGURE 1:**
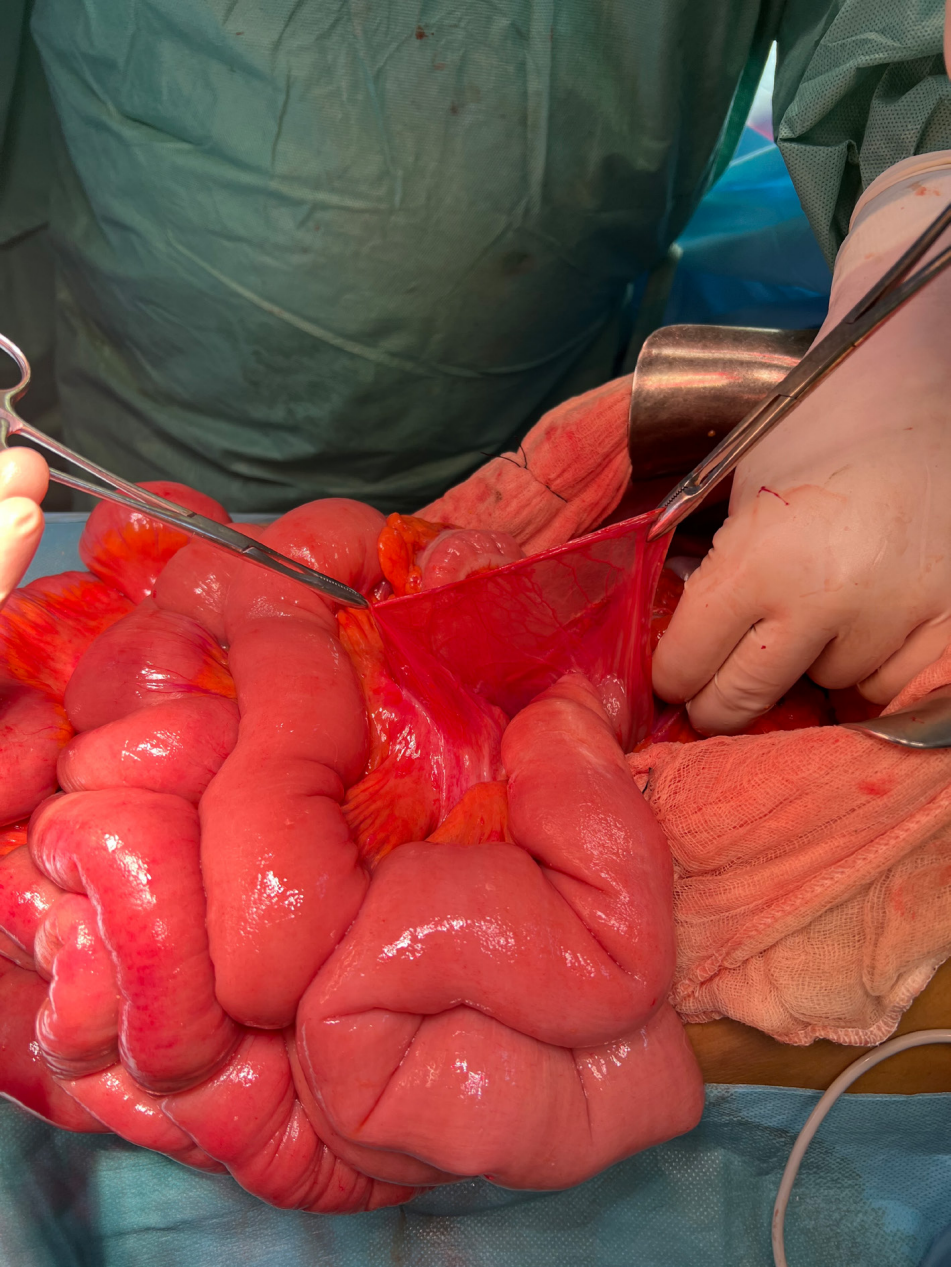
Paraduodenal hernia containing most of the small bowel.

**FIGURE 2:**
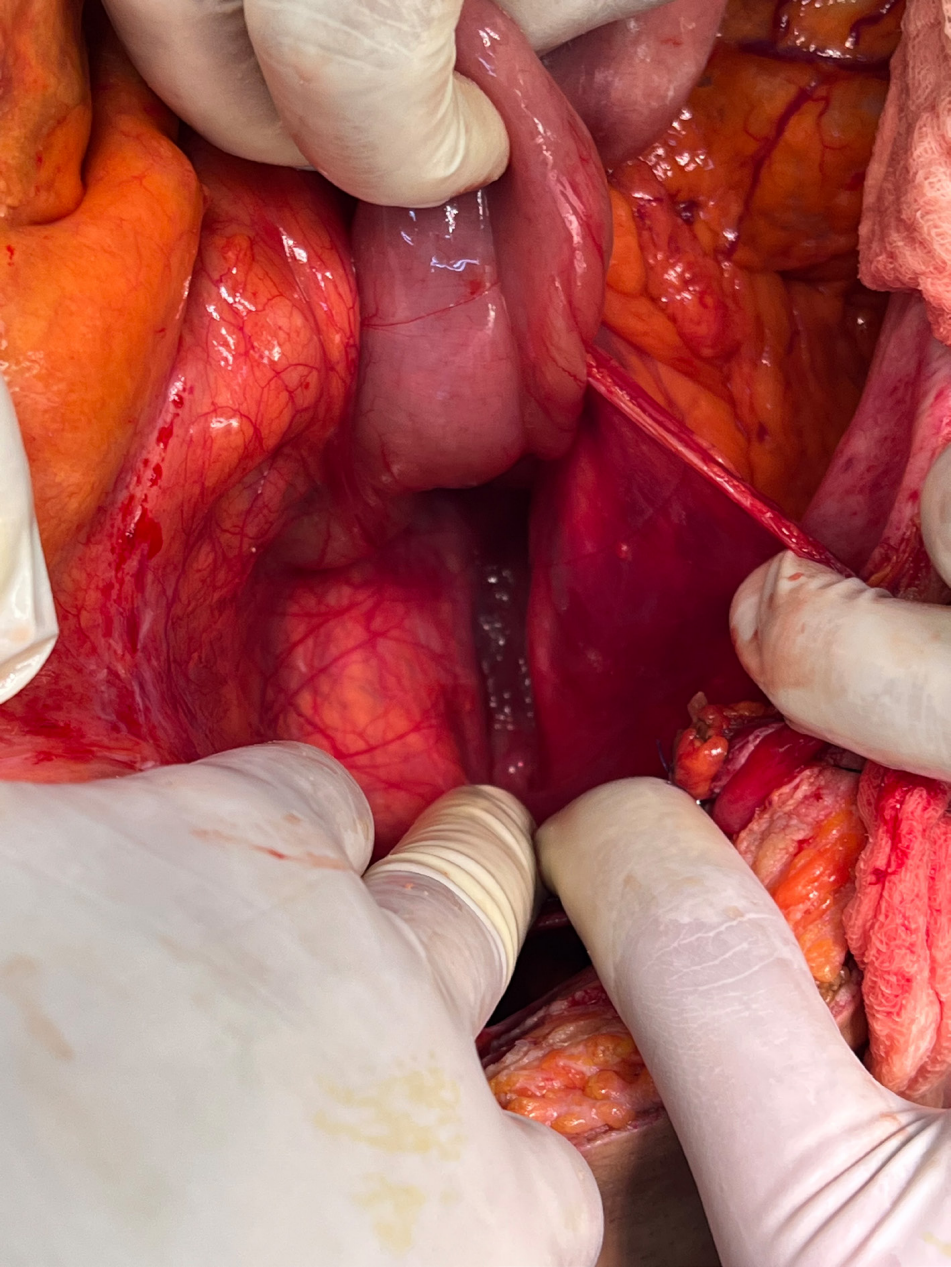
Hernia through a space between the inferior mesenteric vein and duodenojejunal junction.

A 59‐year‐old male patient presented urgently with small bowel obstruction. An abdominal CT scan was performed showing left paraduodenal hernia with an incarcerated jejunal and ileal loops upstream of a single transitional level of the last ileal loop located at the level of the hypogastrium. The diagnosis of acute intestinal obstruction related to paraduodenal hernia was suspected in a virgin abdomen, so we decided to operate the patient. Despite several advantages of laparoscopic approach in such situation, we opted for open surgery. Laparotomy revealed a left paraduodenal hernia (Figure 1) with most of the small bowel herniating through a space between the inferior mesenteric vein and duodenojejunal junction with necking in the last loop (Figure 2). There was no sign of doubtful vitality of small bowel nor effusion. The small bowel was reduced from the hernia, and the defect of the mesentery was repaired using manual sutures with Vicryl.

Paraduodenal hernia (PDH) are responsible for only 0.2%–0.9% of all the cases of intestinal obstruction.^1^ Surgical correction remains the main treatment of PDH. Laparoscopic approach still the gold standard especially in a virgin abdomen as it has several advantages such as decrease in post‐operative pain, reduced morbidity, early food resumption, and shorter hospital stay.^2^ This image has an emphasis on one of the three techniques used for paraduodenal hernia repair: excision of the hernia sac with subsequent closure of the hernia defect.^3^ The other operative approaches are closure or enlargement of defect. However, adopting laparoscopic surgery in such pathology especially in virgin abdomen should promoted more and more.

## AUTHOR CONTRIBUTIONS

**Med Mehdi Trabelsi:** Conceptualization; writing – original draft. **annouar oueslati:** Supervision. **mehdi Kammoun:** Writing – original draft. **hichem jerraya:** Validation; writing – review and editing. **Ibtissem Bouasker:** Validation; writing – review and editing. **Ramzi Nouira:** Validation; writing – review and editing.

## ACKNOWLEDGEMENTS

None.

## CONFLICT OF INTEREST

The authors declare no conflict of interest relevant to this case.

## ETHICAL APPROVAL

Ethics approval and consent to participate. Ethics Committee at Charles Nicolle hospital approved the case study for publication.

## CONSENT

Written informed consent was obtained from the patient to publish this report in accordance with the journal's patient consent policy.

## Data Availability

Data sharing is not applicable.
